# Antiarthritic Effects of a Root Extract from *Harpagophytum procumbens* DC: Novel Insights into the Molecular Mechanisms and Possible Bioactive Phytochemicals

**DOI:** 10.3390/nu12092545

**Published:** 2020-08-23

**Authors:** Alessia Mariano, Antonella Di Sotto, Martina Leopizzi, Stefania Garzoli, Valeria Di Maio, Marco Gullì, Pietro Dalla Vedova, Sergio Ammendola, Anna Scotto d’Abusco

**Affiliations:** 1 Department of Biochemical Sciences, Sapienza University of Roma, P.le Aldo Moro 5, 00185 Roma, Italy; alessia.mariano@uniroma1.it; 2 Department of Physiology and Pharmacology, Sapienza University of Rome, P.le Aldo Moro 5, 00185 Rome, Italy; antonella.disotto@uniroma1.it (A.D.S.); marco.gulli@uniroma1.it (M.G.); 3 Department of Medico-Surgical Sciences and Biotechnologies, Polo Pontino-Sapienza University, 04100 Latina, Italy; martina.leopizzi@uniroma1.it (M.L.); dimaiovaleria@libero.it (V.D.M.); 4 Department of Drug Chemistry and Technologies, Sapienza University of Rome, P.le Aldo Moro 5, 00185 Rome, Italy; stefania.garzoli@uniroma1.it; 5 UOC di Ortopedia e Traumatologia, Ospedale Santa Scolastica di Cassino, ASL di Frosinone, Via S. Pasquale, 03043 Cassino, Italy; pietro.dallavedova@aslfrosinone.it; 6 Ambiotec S.A.S. Via Appia Nord 47, 04012 Cisterna di Latina (LT), Italy; ambiotec@libero.it

**Keywords:** osteoarthritis, nutraceuticals, polyphenols, volatile compounds, β-caryophyllene, eugenol, FAAH, cannabinoid receptors, phospholipases

## Abstract

*Harpagophytum procumbens* (Burch.) DC. ex Meisn. is a traditional remedy for osteoarticular diseases, including osteoarthritis (OA), although the bioactive constituents and mechanisms involved are yet to be clarified. In the present study, an aqueous *H. procumbens* root extract (HPE; containing 1.2% harpagoside) was characterized for its effects on synoviocytes from OA patients and phytochemical composition in polyphenols, and volatile compounds were detected. HPE powder was dissolved in different solvents, including deionized water (HPE_H2O_), DMSO (HPE_DMSO_), 100% *v*/*v* ethanol (HPE_EtOH100_), and 50% *v*/*v* ethanol (HPE_EtOH50_). The highest polyphenol levels were found in HPE_DMSO_ and HPE_EtOH50_, whereas different volatile compounds, mainly β-caryophyllene and eugenol, were detected in all the extracts except for HPE_H2O_. HPE_H2O_ and HPE_DMSO_ were able to enhance CB2 receptor expression and to downregulate PI-PLC β2 in synovial membranes; moreover, all the extracts inhibited FAAH activity. The present results highlight for the first time a multitarget modulation of the endocannabinoid system by HPE, likely ascribable to its hydrosoluble compounds, along with the presence of volatile compounds in *H. procumbens* root. Although hydrosoluble compounds seem to be mainly responsible for endocannabinoid modulation by HPE, a possible contribution of volatile compounds can be suggested, strengthening the hypothesis that the entire phytocomplex can contribute to the *H. procumbens* healing properties.

## 1. Introduction

Osteoarthritis (OA) is a pathology of the whole joint structure, involving several cellular and molecular processes, in different types of cells, such as chondrocytes, osteoblasts, synoviocytes, and immune cells [1]. The clinical symptoms include pain as well as joint dysfunction and deformity, often leading to joint replacement surgery, with high costs for healthcare [2].

Recently, an association between inflammation and endocannabinoid receptors has been described [3]. In particular, the role of CB2 receptors seems very interesting in OA inflammation. CB2 is a peripheral cannabinoid receptor and has been found in immune system cells, raising the possibility that the endocannabinoid system could have a role in immunomodulatory processes [3]. In this respect, several studies reported that mice lacking CB2 receptors showed an exacerbated inflammatory phenotype [4]. Moreover, CB2 receptors are involved also in inhibiting nociceptive transmission [5]. CB2 receptor signaling has been associated with phospholipase C (PI-PLC) activation in calf pulmonary endothelial cells and in mast cells [6,7]. Moreover, the inhibition of inflammatory pathways, such as NF-κB nuclear translocation, has been obtained by inhibiting the PI-PLC β pathway in osteoblast-like cells [8]. The presence of CB2 in articular joints has been explored, mainly in animal models, and it has been highlighted in chondrocytes and in synoviocytes [9,10]. Thus, therapeutic strategies able to modulate CB2 signaling could be considered as a novel approach.

OA is treated with analgesic agents and anti-inflammatory and painkiller drugs, mainly non-steroidal anti-inflammatory drugs (NSAIDs), with the aim of alleviating symptoms [11]. Structure-modifying agents, such as nutraceuticals, are also administered to OA patients, with the aim of preventing or delaying cartilage degradation, even though further studies are required to confirm their effectiveness [12,13,14]. ARTRIT DOL, an Italian food supplement (Italian Minister of Health food supplement no. 71362 since 2013), is a composition containing glucosamine, chondroitin sulfate, extracts from *Harpagophytum procumbens* DC. and *Glycyrrhiza glabra* L., *Curcuma longa* L. roots, manganese, and copper in traces. Although this work does not focus on ARTRIT DOL, it is interesting to note that, as stated by the manufacturer, this nutritional supplement obtained good feedback from OA patients, in particular for the reduction of pain.

*Harpagophytum procumbens* (Burch.) DC. ex Meisn. (Fam. Pedaliaceae), commonly known as devil’s claw, is a plant used worldwide as a traditional remedy for joint pain associated with OA and mild rheumatic ailments [15,16]. Moreover, it has been described to have analgesic effects on neuropathic pain in rats [17]. The harpagoside, one of the characteristic constituents of *H. procumbens* root, has been shown to be effective for osteoarthritis and low back pain [18].

The pharmacological activity of devil’s claw root is attributed to the whole phytocomplex containing iridoid glucosides, such as harpagoside, phenolic glycosides (acteoside and isoacteoside), mono- and polysaccharides, triterpenes (mainly oleanolic acid, 3β-acetyloleanolic acid, and ursolic acid), phytosterols, phenolic acids (caffeic, cinnamic, and chlorogenic acids), flavonoids, and minor components such as volatile compounds [15]. A number of studies have been conducted in order to characterize the analgesic and anti-inflammatory activities of *H. procumbens* and its secondary metabolites. An in vitro study showed that *H. procumbens* was able to decrease the production of proinflammatory cytokines and inhibit metalloprotease activity in human monocytes [19]. Studies conducted in animals showed that an aqueous *H. procumbens* extract showed dose-dependent analgesic and anti-inflammatory activity, but the purified harpagoside did not effectively inhibit the inflammatory pathways, at least at the dosage used in that study [20]. However, human clinical studies showed that the administration of *H. procumbens* root extract was able to improve the clinical picture of OA patients, in terms of pain and limitation of movements [21,22,23], suggesting that the phytocomplex may contribute to observed effects [20]. The biological effects of volatile compounds have not been extensively studied. Recently, the combination of purified β-caryophyllene with curcumin was shown to reduce the inflammation pathway through inhibition of NF-κB in OA primary chondrocytes [24]. Purified β-caryophyllene has been described to mitigate pain in a mouse model of arthritis through a mechanism involving CB2 receptors [25]. Moreover, the volatile eugenol, a known dentistry analgesic, has been shown to be effective in a monoiodoacetate-induced rat model of osteoarthritis [26].

In the present study, an *H. procumbens* root extract was studied for its effects on human primary synoviocytes, with particular attention to the expression of CB2 receptors; furthermore, the extract was characterized for its polyphenol and volatile phytochemical content, in order to highlight possible novel bioactive compounds.

## 2. Materials and Methods

### 2.1. Harpagophytum Procumbens Extract

The dry aqueous extract from *H. procumbens* root (HPE) was provided by Ambiotec S.A.S. It was obtained starting from 300 g of root slices, which were repeatedly washed with cold ethanol 99% (*v*/*v*). After being washed, the slices were reduced by grinding with an electric miller for 10 min, and the powder was recovered by a sieve, with 4 mm holes. This powder was dissolved in ethanol/water solution (1:5 *w*/*v*) in the dark, shaking at 58 °C for 24 h, and then, it was filtered using 0.45 µm membrane. The retained fraction was air dried at 80 °C in a static dryer and in the presence of 5% maltodextrin (as carrier). The powder was again screened by a certified sieve (diameter 200 µm, mesh 100; net light 0.150 µm). The final yield of this preparation was 70% (drug-extract ratio, DER 1.4) of the initial root slices. The extract was a fine brown-colored powder with characteristic smell and taste, containing 1.2% (*w*/*w*) harpagoside. The powder was stored at room temperature in dry and dark conditions until use.

In order to evaluate antiarthritic activity and to highlight possible bioactive constituents, HPE was further dissolved in different solvents, including dimethyl sulfoxide (DMSO), 100% *v*/*v* ethanol (EtOH), 50% *v*/*v* EtOH, and deionized water. These solvents were chosen on the basis of their biocompatibility and ability to dissolve different classes of phytochemicals, mainly focusing on volatile compounds and polyphenols. Particularly, DMSO possesses high solubilizing properties for both polar and nonpolar compounds, thus, being able to dissolve the entire phytocomplex. Conversely, deionized water and ethanol recover mainly polar and nonpolar molecules, respectively, whereas 50% *v*/*v* ethanol is able to collect compounds dissolved by both solvents.

### 2.2. Phytochemical Analysis

#### 2.2.1. Determination of Total Polyphenols, Tannins, and Flavonoids

Total amounts of polyphenols and tannins in the tested extracts were determined spectrophotometrically by the Folin–Ciocalteu method, as previously reported [27]. For both polyphenols and tannins, the absorbance was measured at 765 nm, and the amount was calculated as tannic acid equivalent (TAE) per milligram of dry HPE extract. Furthermore, total flavonoids and its subclass flavonols were measured by applying the aluminum chloride method with minor changes [27]. Specifically, total flavonoids were measured after mixing equal volumes of each extract (2 mg/mL) and aluminum trichloride (2% *w*/*v* in methanol), whereas the content of flavonols was determined by mixing 50 μL extract (4 mg/mL), 20 μL aluminum trichloride (10% *w*/*v* in methanol), 60 μL sodium hydroxide (1 M), 10 μL sodium nitrite (5% *w*/*v* in deionized water), and 70 μL deionized water. After a 10-min incubation, the absorbance was measured at 415 nm, and the total contents of flavonoids and flavonols were determined and expressed as quercetin equivalent (QE) per milligram of dry HPE extract.

#### 2.2.2. Solid-Phase Microextraction (SPME)

For the extraction of volatile compounds, solid-phase microextraction (SPME) holders and coating fibers (Supelco; Bellefonte, PA, USA) were used. The sampling was performed with an SPME fiber (50/30 μm divinylbenzene/carboxen/polydimethylsiloxane—DVB/CAR/PDMS). Before sampling, the SPME fiber was conditioned by heating in the injector of a gas chromatograph at 270 °C for 30 min in order to remove traces of contaminants. Prior to analysis, a fiber blank was run to confirm the absence of contaminant peaks. To obtain a better extraction, SPME conditions, such as the most suitable temperature and equilibration time, were adjusted. Each sample (0.5 mL) was placed in a septum-sealed glass vial. The fiber was exposed to the headspace of the sample for 20 min at 40 °C. During this time, samples were stirred with a magnetic stirrer. After equilibration, the fiber was removed from the sample and immediately inserted into the GC injection port for the thermal desorption (2 min) at 270 °C.

#### 2.2.3. Gas Chromatography–Mass Spectrometry (GC–MS)

The extracts were analyzed using a GC–MS Perkin Elmer Clarus 500 instrument (Perkin Elmer, Waltham, MA, USA) equipped with a flame ionization detector (FID). Chromatographic separations were performed on a Varian FactorFour VF-1 fused-silica capillary column (length 60 m × 0.32 mm ID × 1.0 μm film thickness). The oven temperature program was as follows: 60 °C for two min, then, a gradient of 6 °C/min to 250 °C for 10 min, and an injector temperature of 270 °C. Helium was used as the carrier gas with a flow rate of 1.0 mL/min. Split injection with a split ratio of 1:20 was used. The electron-impact ionization mass spectrometer was operated as follows: ionization voltage, 70 eV; ion source temperature, 200 °C; scan mode, 30.0 to 500.0 mass range. The volatile compounds were identified by comparing mass spectra with those in the NIST02 and Wiley libraries. Furthermore, linear retention indices (LRIs) of each compound were calculated using a mixture of n-alkane hydrocarbons (C8-C30, Ultrasci, Ultra Scientific Italia, Bologna, Italy) injected directly into GC injector using the same temperature program reported above. Semiquantitative analysis was performed by normalizing the peak area generated in the FID (%) without using correction factors (relative response factors, RRFs). All analyses were repeated twice.

### 2.3. Human Tissue

Human synovial membranes were isolated from 6 OA patients and 5 non-OA (fractured) patients that underwent surgical treatment. Full ethical consent was obtained from all donors and the Research Ethics Committee, Sapienza University of Roma (#290/07, 29 March 2007), and ASL Lazio 2 (#005605/2019, 3 March 2019) approved the study. The tissues were fixed in 4% paraformaldehyde in 0.1 M phosphate buffer pH 7.2 immediately after removal from patients.

### 2.4. Immunohistochemistry

Histological sections were deparaffinized and rehydrated in graded ethanol. Endogenous peroxidase activity was blocked by 3% hydrogen peroxide for 10 min. Antigen retrieval was performed in 10 mM sodium citrate buffer (pH 6.0) for 15 min. The sections were then incubated with anti-CB1, anti-CB2, anti-PI PLC β2, and anti-PI PLC β3 (Santa Cruz Biotechnology, Inc., Dallas, TE, USA), all diluted 1:50, overnight at 4 °C. After incubation, specimens were washed and incubated with the secondary-biotinylated antibody and subsequently, with streptavidin–biotin–peroxidase (DAKOLSAB Kit peroxidase; DAKO, Carpinteria, CA, USA). The signals were developed by incubating with freshly prepared 3,3′-diaminobenzidine (DAB) substrate–chromogen buffer at room temperature. Slides were counterstained with hematoxylin and mounted with permanent mounting media. Negative controls were used in each experiment. The samples were scored semiquantitatively using a score based on the intensity and distribution: 0, undetectable; 1+, weak staining; 2+, medium staining; 3+, strong staining [28].

### 2.5. Human Primary Cell Isolation

Human primary synoviocytes (FLSs) were isolated from synovial membranes, obtained, as above described, from patients who underwent a total knee and hip arthroplasty. In brief, the synovial membrane fragments were minced and treated with 1 mg/mL collagenase type IV and 0.25% trypsin for 1 h, at 37 °C in agitation. Then, FLSs were grown to 80% confluence in DMEM (HyClone, Logan, UT, USA) supplemented with L-glutamine, penicillin/streptomycin (Sigma-Aldrich, St. Louis, MO, USA), and 10% fetal bovine serum (FBS) and cultured at 37 °C and 5% CO_2_. All experiments were carried out with synoviocytes at first passage (p1), isolated from at least 3 different donors.

### 2.6. Immunofluorescence

Vimentin, CB2, and PI-PLC β2 were visualized by immunofluorescence. Cells were plated at a density of 8 × 10^3^/cm^2^ and cultured for 48 h and then, washed in PBS, fixed in 4% paraformaldehyde in PBS for 15 min at 4 °C, and permeabilized with 0.5% Triton-X 100 in PBS for 10 min at room temperature. After blocking with 3% bovine serum albumin (BSA) in PBS for 30 min at room temperature, cells were incubated at 1 h, at room temperature, with mouse monoclonal anti-vimentin antibody (Proteintech Group, Manchester, UK) 1:50, mouse monoclonal anti-CB2 antibody 1:150, and mouse monoclonal anti-PI-PLC β2 antibody (Santa Cruz Biotechnology) 1:50. Cells were washed with PBS and then, incubated for 1 h, at room temperature, with Alexa Fluor 488 donkey anti-rabbit antibody 1:300 (Invitrogen, Thermo Fisher Scientific, Waltham, MA, USA), to stain vimentin green; with Alexa Fluor 488 donkey anti-goat antibody 1:600 (Invitrogen, Thermo Fisher Scientific), to stain CB2 receptors green; and Alexa Fluor 595 donkey anti-rabbit antibody 1:300 (Invitrogen, Thermo Fisher Scientific), to stain PI-PLC β2 red. Slides were washed and then, stained with DAPI (Invitrogen, Thermo Fisher Scientific) to visualize the nuclei. The images were captured by a Leica DM IL LED optical microscope, using an AF6000 modular microscope (Leica Microsystem, Milan, Italy).

### 2.7. Densitometric Analysis

The free software ImageJ (https://imagej.nih.gov/ij/) was used to perform the densitometric analysis of protein production. For each cell culture condition, the integrated density values of fluorescence were considered.

### 2.8. Cell Treatment

Cells were left untreated (CTL) or treated, for the required time, with 0.1 mg/mL of *Harpagophytum procumbens* root extract (HPE) dissolved in deionized water (HPE_H2O_), DMSO (HPE_DMSO_), 100% *v*/*v* EtOH (HPE_EtOH100_), and 50% *v*/*v* EtOH (HPE_EtOH50_). Experiments were independently repeated at least three times.

### 2.9. Cell Viability

To assess a potential cytotoxic effect of *H. procumbens* extracts on FLSs at different concentrations and time points, an MTS (3-(4,5-dimethylthiazol-2–yl)-5-(3-carboxymethoxyphenyl)-2-(4-sulfophenyl)-2*H*-tetrazolium)-based colorimetric assay was performed (Promega Corporation, Madison, WI, USA). Briefly, 8 × 10^3^ cells per well were seeded in a 96-well plate. The day after seeding, cells were starved overnight in reduced serum medium, in order to align cell cycle progression. Cells were then left untreated (CTL) or treated with *H. procumbens* extracts for 24, 48, and 72 h. After each time point, 100 μL MTS solution was added to the wells. Spectrophotometric absorbance was directly measured at 492 nm after 3 h incubation.

### 2.10. RNA Extraction and Reverse Transcription

Total RNA was extracted with TRIZOL (Invitrogen, Thermo Fisher Scientific), purified using a micro RNeasy column (Qiagen, Valencia, CA, USA), and reverse transcribed by Improm II enzyme, (Promega Corporation, Madison, WI, USA), according to the manufacturers’ instructions.

### 2.11. Quantitative Real-Time PCR

Quantitative real-time PCR analysis was performed using an ABI Prism 7300 (Applied Biosystems, Thermo Fisher Scientific). Amplification was carried out using SensimixPlus SYBR master mix (Bioline, London, UK). Primers were designed using Primer Express software (Applied Biosystems) and were synthesized by Biofab Research (Rome, Italy). Primers’ sequences are reported in Table 1.

Data were analyzed by the 2^−ΔΔCt^ method, which determines the transcript abundance relative to the 18S housekeeping gene [29].

### 2.12. FAAH Inhibition

The potential ability of the tested extract to inhibit fatty acid amide hydrolase (FAAH) was evaluated using a commercial fluorescence-based kit (Cayman’s FAAH Inhibitor Screening Assay Kit, Vinci Biochem, Vinci (FI), Italy), according to the manufacturer’s instructions. The fluorescence of the FAAH-catalyzed product was measured at an excitation wavelength of 340 to 360 nm and an emission wavelength of 450 to 465 nm by a BD Accuri™ C6 flow cytometer (BD Biosciences, Milan, Italy). Suitable control wells treated with vehicles (maximum FAAH activity) and with the known FAAH inhibitor JZL 195 (maximum FAAH inhibition) were included. Each treatment was assayed at least in triplicate and at least in two different experiments. The enzyme activity was evaluated as % inhibition with respect to the vehicle.

### 2.13. Statistical Analysis

All data were obtained from at least three independent experiments, each performed either in duplicate or in triplicate (n = 6 or n = 9). Data were statistically analyzed with two-way repeated measures analysis of variance (ANOVA) with Bonferroni’s multiple comparison test using Prism 5.0 software (GraphPad Software, San Diego, CA, USA). The *p* value < 0.05 was considered significant.

The Hill equation E = E_max_/(1 + (10^LogEC50/A^) HillSlope) (E, effect at a given concentration; E_max_, maximum activity; EC_50_ or IC_50_, concentration giving a 50% inhibition; A, concentration of agonist; HillSlope, slope of the agonist curve) was applied to obtain a concentration–response curve.

## 3. Results

### 3.1. Phytochemical Characterization

The highest levels of total polyphenols were extracted by DMSO and 50% *v*/*v* EtOH, followed by deionized water and pure ethanol. Indeed, their levels in HPE_DMSO_ and HPE_EtOH50_ were 1.2- to 1.4-fold higher than in HPE_H2O_ and HPE_EtOH100_ (Table 2). Tannins were mainly recovered by H_2_O and DMSO, their amount in HPE_H2O_ and HPE_DMSO_ being 1.5- to 1.9-fold higher than that found in HPE_EtOH100_ and HPE_EtOH50_ (Table 2). Regarding flavonoids and flavonols, the highest extraction power was exhibited by DMSO; HPE_DMSO_ exhibited about 1.5- to 3-fold and 1.5- to 1.7-fold higher concentrations of total flavonoids and flavonols than HPE_EtOH50_ or HPE_EtOH100_ and HPE_H2O_, respectively (Table 2).

Altogether, these results revealed that DMSO is the most suitable for recovering total polyphenols, including tannins, flavonoids, and flavonols, followed by 50% *v*/*v* EtOH, especially for total flavonoids and flavonols. Pure ethanol extract possesses similar features compared to 50% *v*/*v* EtOH regarding total flavonoids and flavonols, with a lower recovery of total polyphenols. Finally, pure deionized water was able to better extract tannins, with significantly lower flavonoid levels.

The SPME–GC–MS analysis highlighted the presence of different volatile compounds in all the samples except for the aqueous HPE_H2O_ extract. Among them, eight compounds, listed in Table 3, were identified and their relative percentage amounts were calculated. Eugenol and β-caryophyllene were the main volatile phytochemicals in all extracts. Eugenol achieved a maximum 51.6% amount in HPE_ETOH50_, while HPE_DMSO_ and HPE_ETOH100_ contained the highest percentages of β-caryophyllene (i.e., 77.4% and 77.1%, respectively). Moreover, β-pinene (5.1%) and isoeugenol (1.0%) were found in HPE_ETOH50_, while thymol (0.8%) was found in HPE_DMSO_. α-copaene was also identified in ethanolic extracts HPE_EtOH100_ and HPE_EtOH50_ (6.3% and 3.7%, respectively). On the other hand, α-humulene (10.0%; 0.8%) and δ-cadinene (5.8%; 1.3%) were detected in HPE_DMSO_ and HPE_EtOH100_, respectively.

### 3.2. Expression of Cannabinoid Receptors in OA Synovial Membrane

**Figure 1:**
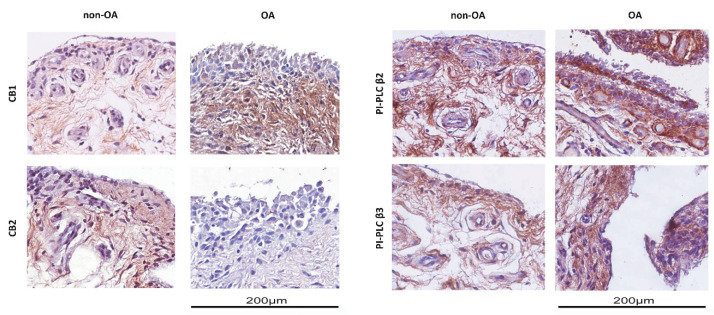
Immunohistochemical analysis of synovial membranes from OA (osteoarthritis) and non-OA patients. Left panel: Slices were stained with anti-CB1 and anti-CB2 receptor antibodies. Right panel: Slices were stained with anti-PI-PLC β2 and anti-PI-PLC β3 antibodies. Slides were counterstained with hematoxylin and mounted with permanent mounting media. This figure shows representative images of different experiments (n = 5 non-OA and n = 6 OA).

The presence of CB1 and CB2 receptors and PI-PLC β2 and β3 was detected by immunohistochemistry in synovial membranes isolated both from non-OA and OA patients. CB1 receptors were expressed in both normal and pathological tissues but with different scores; they were moderately present in non-OA patients (score 1+), while strongly present in OA patients (score 3+) (Figure 1, left-upper panel). CB2 receptors were detected only in non-OA tissue (score 2+) (Figure 1, left-bottom panel). Regarding the presence of PI-PLC β2 and β3, we found that the expression of PI-PLC β2 showed a lesser staining in non-OA (score 2+) than OA tissue (score 3+), and it was localized in the cytoplasm of both tissues (Figure 1, right-upper panel). PI-PLC β3 was equally expressed in tissues from non-OA (score 3+) and OA patients (score 3+) (Figure 1, right-bottom panel). We also analyzed PI-PLC β1 and PI-PLC β4, finding that were both barely expressed both in non-OA and OA synovial membranes.

### 3.3. Effects of Harpagophytum Extracts on Synoviocyte Cell Viability

**Figure 2:**
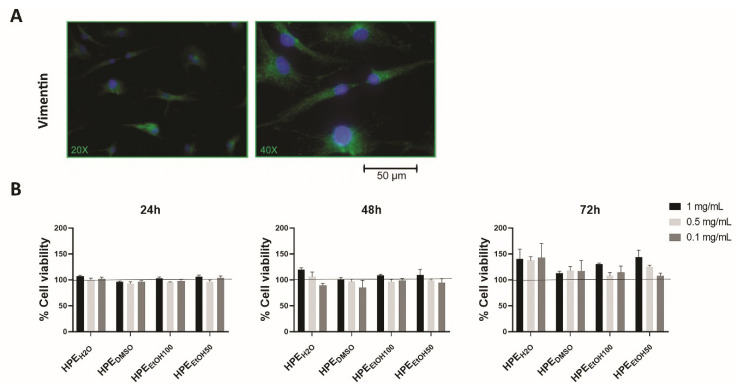
Characterization of human primary synoviocytes (FLSs) and analysis of cell viability. (**A**) Human primary FLSs, isolated by synovial membranes and cultured in vitro, were stained with anti-vimentin primary antibody and with Alexa Fluor 488 (green) secondary antibody. (**B**) Cell viability was assessed by the MTS colorimetric method, and FLSs were treated with three concentrations, 1, 0.5, and 0.1 mg/mL of *Harpagophytum procumbens* root extract (HPE) dissolved in deionized water (HPE_H2O_), DMSO (HPE_DMSO_), 100% *v*/*v* EtOH (HPE_EtOH100_), and 50% *v*/*v* EtOH (HPE_EtOH50_), for 24, 48, and 72 h. Cell viability of treated samples was normalized to the untreated cells, which is reported as 100% and represented by a horizontal line.

Synoviocytes were isolated by both OA and non-OA tissues. Two types of synoviocytes are present in the synovial membrane, A and B. The synoviocytes A are macrophage-like and the synoviocytes B are fibroblast-like synoviocytes (FLSs). The latter are characterized by their elongated shape and the expression of vimentin [30]; we verified the expression of this protein in our cells, finding that they were able to produce vimentin (Figure 2A).

The effects of *Harpagophytum procumbens* extracts on FLS cell viability were determined by the MTS colorimetric method. The extracts, tested at 1 mg/mL, 0.5 mg/mL, and 0.1 mg/mL for 24, 48, and 72 h, did not show detrimental effects at any analyzed concentration or time point (Figure 2B). We decided to use the 0.1 mg/mL concentration for further experiments.

### 3.4. Mechanism of Action of Harpagophytum Extracts on FLSs

**Figure 3:**
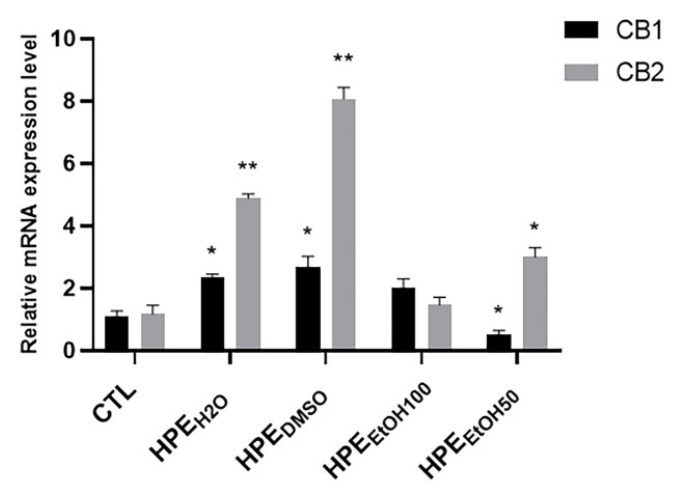
Effects of all HPE extracts on CB1 and CB2 mRNA expression level in human primary FLSs. After 24 h treatment with 0.1 mg/mL of *Harpagophytum procumbens* root extract (HPE) dissolved in deionized water (HPE_H2O_), DMSO (HPE_DMSO_), 100% *v*/*v* EtOH (HPE_EtOH100_), and 50% *v*/*v* EtOH (HPE_EtOH50_), cells were harvested and mRNA was extracted and analyzed by RT-PCR. CB1 and CB2 receptor mRNA levels were reported as relative mRNA expression level with respect to 18S mRNA (2^−ΔΔCt^ method). Results are expressed as mean ± S.E.M. of data obtained by three different experiments. Statistical significance was * *p* < 0.05; ** *p* < 0.01.

In order to assay the effects of the different extracts on cannabinoid receptor expression, they were added to cell culture medium at a concentration of 0.1 mg/mL for 24 h, then, the mRNA expression level of CB1 and CB2 receptors was analyzed. The HPE_H2O_ and HPE_DMSO_ and to a lesser extent, HPE_EtOH50_, were able to increase the CB2 mRNA expression level, whereas HPE_EtOH100_ did not show any effect (Figure 3). CB1 receptor mRNA expression level was increased by HPE_H2O_ and HPE_DMSO_ and to a lesser extent by HPE_EtOH100_, whereas it was decreased by HPE_EtOH50_ (Figure 3).

**Figure 4:**
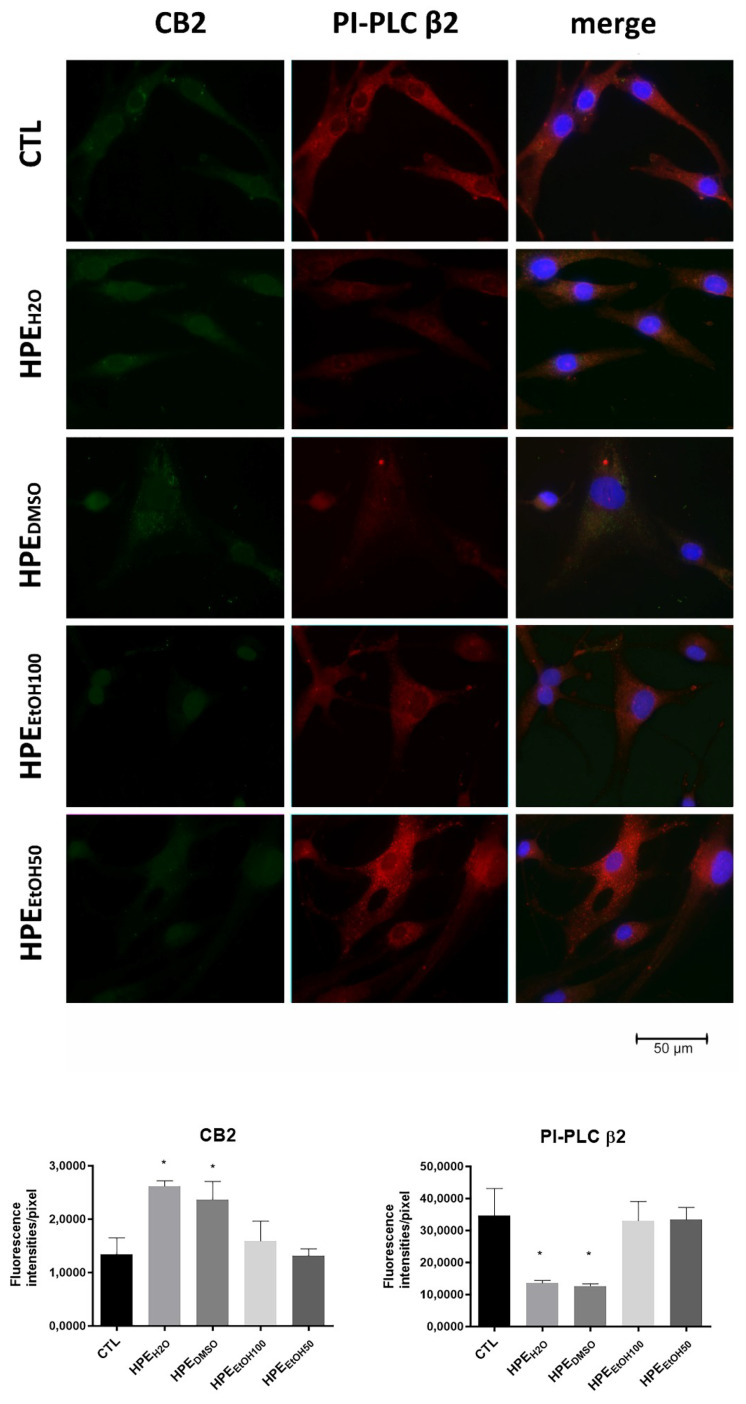
Effects of all HPE extracts on CB2 receptor and PI-PLC β2 protein production. Upper panel: Cells were treated with 0.1 mg/mL of *Harpagophytum procumbens* root extract (HPE) dissolved in deionized water (HPE_H2O_), DMSO (HPE_DMSO_), 100% *v*/*v* EtOH (HPE_EtOH100_), and 50% *v*/*v* EtOH (HPE_EtOH50_), for 24 h and then, analyzed by immunofluorescence using anti-CB2 and anti-PI-PLC β2 primary antibodies and Alexa Fluor 488 (green, CB2) and Alexa Fluor 568 (red, PI-PLC β2) secondary antibodies, respectively. Nuclei were stained with DAPI (original magnification 40×). Lower panel: The pixel intensities in the region of interest were obtained by ImageJ. * *p* < 0.05.

Considering that only CB2 receptors are associated with inflammation and pain in peripheral tissues, we verified whether the CB2 receptors also increased at the protein level by immunofluorescence staining. FLSs were treated with 0.1 mg/mL extracts for 24 and 48 h, then, the cells were stained with antibody anti-CB2. We found that HPE_H2O_ and HPE_DMSO_ extracts were able to stimulate the exposure in membrane of CB2 receptors, both at 24 and 48 h, whereas HPE_EtOH50_ and HPE_EtOH100_ did not stimulate CB2 receptor expression at any analyzed time (Figure 4 and Figure S1). Taking into account that the expression of PI-PLC β2 was increased in synovial membranes from OA patients, we verified whether the HPE extracts were able to reduce the production of this phospholipase. HPE_H2O_ and HPE_DMSO_ were able to inhibit the expression of PI-PLC β2, whereas HPE_EtOH50_ and HPE_EtOH100_ did not show effects (Figure 4).

### 3.5. Inhibition of Fatty Acid Anandamide Hydrolase

**Figure 5:**
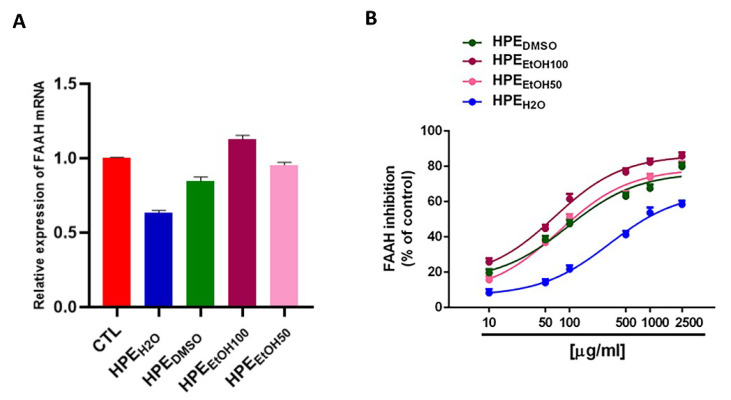
Effects of *Harpagophytum procumbens* root extract (HPE) on fatty acid anandamide hydrolase (FAAH) expression and enzymatic activity. (**A**) Cells were treated with 0.1 mg/mL of *Harpagophytum procumbens* root extract (HPE) dissolved in deionized water (HPE_H2O_), DMSO (HPE_DMSO_), 100% *v*/*v* EtOH (HPE_EtOH100_), and 50% *v*/*v* EtOH (HPE_EtOH50_), for 24 h. Cells were then harvested, and mRNA was extracted and analyzed by RT-PCR. FAAH mRNA levels were reported as relative mRNA expression level with respect to 18S mRNA (2^−ΔΔCt^ method). Results are expressed as mean ± S.E.M. of data obtained by three different experiments. (**B**) Concentration–response curves showing the inhibitory effects on FAAH from HPE_DMSO_, 100% *v*/*v* HPE_EtOH100_, 50% *v*/*v* HPE_EtOH50_, and HPE_H2O_. Data are the mean ± SE of at least three independent experiments with two replicates for each experiment (n = 6).

In order to check whether the HPE extracts were able to affect the fatty acid amide hydrolase (FAAH), we analyzed both the FAAH mRNA expression level and enzymatic activity. HPE_H2O_ was able to decrease the FAAH mRNA level even if the downregulation was not statistically significant, whereas all other extracts were ineffective (Figure 5A). Interestingly, under our experimental conditions, all the extracts were able to interfere with the FAAH activity, although with different efficacy and potency (Figure 5B). Particularly, HPE_H2O_ was the least effective sample, achieving a maximum 58.5% enzyme inhibition at the highest concentration of 2500 μg/mL. Conversely, the other extracts could completely inhibit the FAAH enzyme, HPE_EtOH100_ being slightly more potent than HPE_DMSO_ and HPE_EtOH50_, which displayed similar potencies. Indeed, the IC_50_ value of HPE_EtOH100_ was about 1.2- to 1.5-fold lower than those of HPE_EtOH50_ and HPE_DMSO_ (Table 4). Under the same experimental conditions, 100 μg/mL harpagoside (corresponding to 200 μM) was found to be ineffective in the inhibition of the FAAH enzyme (about 9% inhibition compared the control). Conversely, the positive control JZL 195 (20 μM corresponding to 8.7 μg/mL) produced a maximum 90% enzyme inhibition (Table 4). As expected, the positive control was significantly more potent than the HPE extracts (Table 4).

## 4. Discussion

The aim of this study was to investigate the effects of *Harpagophytum procumbens* extract (HPE) on fibroblast-like synoviocytes (FLSs) from osteoarthritis patients, with particular attention on the endocannabinoid-mediated mechanisms. Osteoarthritis (OA) is characterized by chronic inflammation, and it is currently treated with anti-inflammatory drugs, which are only able to counteract the symptoms [2,31]. Several nutraceuticals, such as glucosamine, chondroitin sulfate, and curcumin, are administered with the aim of delaying the cartilage degradation, with inconsistent results [32]. Extracts from plants are also traditionally used to treat OA [16]. Among them, *H. procumbens* DC. has been studied for its chondroprotective activities, mainly for the ability to inhibit the production of proinflammatory mediators, such as TNFα and IL-1β, and enzymes able to hydrolyze the extracellular matrix components, metalloproteases, and elastase [19].

Iridoid glycosides have received major attention as possible bioactive compounds of *H. procumbens* secondary metabolites [33]. Harpagoside is the most investigated one, and it is considered a reference standard of *H. procumbens* for titration purposes [15]. The anti-inflammatory activity of harpagoside has been found to be mediated by the inhibition of COX-1 and COX-2 enzymes along with by a lowered cytokine and NO release [33]. It was also able to counteract inflammation in primary human osteoarthritic chondrocytes through the suppression of c-FOS/AP-1 signal and the inhibition of proinflammatory cytokine and fibrinogenic factor production [23]. Moreover, harpagoside requires hydrolysis to a bioactive metabolite to exert its anti-inflammatory activity [34].

Despite this promising evidence, the anti-inflammatory properties of harpagoside cannot fully explain those of the entire *H. procumbens* phytocomplex, thus, suggesting that other compounds can contribute to the activity of the plant [15,33]. In the phytocomplex, some phenylpropanoids were reported to contribute to the *H. procumbens* anti-inflammatory effects [15].

Some studies have highlighted that plant roots can release volatile compounds as a defense strategy to counteract pathogen and fungal infections and to mediate the interaction between plant and soil bacteria [35,36,37]. Accordingly, our SPME–GC–MS analysis of HPE extracts highlighted the presence of several volatile compounds, mainly recovered by HPE_DMSO_, 100% *v*/*v* HPE_EtOH_, and 50% *v*/*v* HPE_EtOH_. Among these volatile compounds, β-caryophyllene and eugenol were identified to be the major sesquiterpene and monoterpene present, followed by the sesquiterpenes α-humulene and α-copaene. Previously, 31 different volatile compounds, obtained by an heptanoic extraction, were characterized in *H. procumbens* root, although neither sesquiterpenes nor monoterpenes were detected [38]. In several preclinical models, β-caryophyllene, α-humulene, and eugenol have been shown to possess anti-inflammatory activities, affecting proinflammatory cytokine secretion and inhibiting inducible nitric oxide synthase (iNOS) and cyclooxygenase (COX-2) expression [39,40]. β-caryophyllene acts as an agonist of the endocannabinoid CB2 receptor, which is involved in the modulation of inflammation and the immune system and inhibits the fatty acid amide hydrolase enzyme [41,42]. Recent evidence highlighted the ability of this sesquiterpene to reduce articular and systemic inflammation in several animal models of arthritis [25,43,44]. Particularly, its antiarthritic effects in human articular chondrocytes were mediated by a crosstalk between CB2 and PPAR-γ receptors [25].

In articular joints, FLSs are involved both in supporting health chondrocytes and the immune system during inflammation [45]. In this study, we decided to use FLSs isolated from human synovial membranes as an in vitro model to study the effects of *H. procumbens* extracts. Preliminarily, the expression of CB2 receptors was checked in synovial membranes from OA and non-OA joints, and we found that it was expressed only in non-OA joints and was completely absent in OA. This finding agrees with the observations of Fukuda and coworkers, who found that CB2 receptors are expressed in rheumatoid arthritis (RA) synovial membranes and not in OA synovial membranes [46]. Moreover, they showed that CB2 plays an anti-inflammatory role in RA, and the treatment of RA synoviocytes with an agonist of CB2 blocked the production of proinflammatory mediators, through the inhibition of adenylyl cyclase, which, in turn, did not produce cAMP. Thus, protein kinase A was not activated, finally leading to failure of the activation of NF-κB [46]. Accordingly, the absence of CB2 in OA synovial membranes, which has been observed in our samples, can be associated with the activated inflammatory pathways present in joints of OA patients. Several studies showed the activation of NF-κB in OA joints [34,47]; thus, the activation of pathways or molecules that can downregulate NF-κB activity is very desirable [48,49]. Moreover, Sophocleous and coworkers showed that CB2^-/-^ mice had a greater susceptibility to OA [4].

The phosphoinositide (PI)-dependent signal plays important roles in many cellular processes, among them proinflammatory pathways, the alteration of which is involved in the onset and progression of several diseases [50]. The PI-phospholipase C (PI-PLC) β1 isoform has been described as differently modulated in osteoblasts from OA and RA patients [51]. We analyzed the synovial membranes from OA and non-OA patients for the presence of PI-PLC β1–β4, finding that β1 and β4 were almost unexpressed both in OA and non-OA tissues, whereas β3 was equally expressed in both tissues. Interestingly, β2 was highly expressed in OA and poorly expressed in non-OA, suggesting that only β2 is related to injured OA tissue. These findings prompted us to evaluate whether the *H. procumbens* extracts were able to modulate both CB2 and PI-PLC β2 in isolated human primary FLSs, finding that HPE_H2O_ and HPE_DMSO_ stimulated the expression of CB2 receptors and decreased the expression of PI-PLC β2, whereas the HPE_EtOH50_ had a weak effect and HPE_EtOH100_ was completely ineffective on the expression of these two molecules. Thus, the ability of HPE_H2O_ and HPE_DMSO_ to increase the expression of CB2 receptors may explain the anti-inflammatory and antinociceptive activity of this plant. The role of PI-PLC β2 in inflammatory pathways has not been described so far; for this reason, the inhibition of its expression needs to be explored more in depth. We suppose that its expression in OA tissue could be associated with stimulation of the proinflammatory pathway through the activation of protein kinase C.

Moreover, further anti-inflammatory mechanisms are affected, as shown by the inhibition of FAAH in all the analyzed HPE extracts. The best inhibition was shown by HPE_EtOH100_ and HPE_EtOH50_, so we can hypothesize that volatile compounds, such as β-caryophyllene, eugenol, and α-humulene, can be involved in these HPE effects.

## 5. Conclusions

Harpagoside, harpagide, and procumbide are present in all extracts, and ethnopharmacology considers them primarily responsible for *H. procumbens* activity. However, several studies, performed at the molecular level, showed that the administration of these purified compounds cannot justify the analgesic activity of the whole phytocomplex. The present study showed that further extractions with deionized water or DMSO can recover some bioactive compounds able to increase the synthesis of CB2 receptors, which are unexpressed in the osteoarthritic tissues. Moreover, the deionized water and DMSO extracts decrease the expression of the PI-PLC β2 isoform, which, in turn, could inhibit the FAAH synthesis of endocannabinoids. Interestingly, DMSO, 50% ethanolic, and to a greater extent, 100% ethanolic extracts, were able to inhibit FAAH activity.

Further studies in in vitro cell models and in vivo animal models are needed to support the hypothesis that *H. procumbens* root can be effective in controlling osteoarthritic pain, through the modulation of endocannabinoid system. The specific contribution of iridoid glucosides, polyphenols, and terpenes deserve to be further investigated too.

## Figures and Tables

**Table 1:** Sequences of the primers used in RT-PCR analysis.

Gene	Primer Sequences
**CB1** **NM_016083**	Forward: 5′-TTCCTTCTTGTGAAGGCACTG-3′ Reverse: 5′-TCTTGACCGTGCTCTTGATGC-3′
**CB2** **NM_001841**	Forward: 5′-ATGCTGTGCCTCATCAACTC-3′ Reverse: 5′-CTCACACACTTCTTCCAGTG-3′
**FAAH** **NM_001441**	Forward: 5′-CAGCTTTCCTCAGCAACATG-3′ Reverse: 5′-CAATCACGGTTTTGCGGTAC-3′
**18S** **NM_003286**	Forward: 5′-CGCCGCTAGAGGTGAAATTC-3′ Reverse: 5′-CATTCTTGGCAAATGCTTTCG-3′

**Table 2:** Amounts of total polyphenols, tannins, flavonoids, and flavonols in the extract from *Harpagophytum procumbens* root (HPE) dissolved in DMSO (HPE_DMSO_), 100% *v*/*v* EtOH (HPE_EtOH100_), 50% *v*/*v* EtOH (HPE_EtOH50_), and deionized water (HPE_H2O_). Data are the mean ± SE of at least three independent experiments with three replicates for each experiment (n = 9).

Compounds	HPE_DMSO_	HPE_EtOH100_	HPE_EtOH50_	HPE_H2O_
	µg/mg of Dry Extract (Mean ± SE)			
Polyphenols (TAE)	99.6 ± 0.05 ***^§^	69.6 ± 0.05	97.9 ± 0.01 ***^§^	84.2 ± 0.04 **
Tannins (TAE)	14.2 ± 0.02 **	9.2 ± 0.04	9.2 ± 0.02	17.9 ± 0.03 ***
Flavonoids (QE)	113.0± 5.1 ***^§§^	71.3 ± 6.6 ^§§^	75.1 ± 6.1 ^§§^	37.6 ± 4.3
Flavonols (QE)	43.8 ± 2.4 ***^§§^	27.6 ± 3.2	30.1 ± 2.0 ^§^	25.1 ± 2.0

TAE—tannic acid equivalent. QE—quercetin equivalent. ** *p* < 0.01 and *** *p* < 0.01 significantly higher than HPE_EtOH100_ (ANOVA followed by Bonferroni’s multiple comparison post hoc test). ^§^ *p* < 0.05 and ^§§^ *p* < 0.01 significantly higher than HPE_H2O_ (ANOVA followed by Bonferroni’s multiple comparison post hoc test).

**Table 3:** Volatile compounds (relative percentage in the volatile fraction) detected in *Harpagophytum procumbens* root extract (HPE) dissolved in DMSO (HPE_DMSO_), 100% *v*/*v* EtOH (HPE_EtOH100_), 50% *v*/*v* EtOH (HPE_EtOH50_), and deionized water (HPE_H2O_).

No. ^1^	Compound ^2^	LRI ^3^	LRI ^4^	MS ^5^	HPE_DMSO_ (%)	HPE_EtOH100_ (%)	HPE_EtOH50_ (%)	HPE_H2O_ (%)
1	β-pinene	978	974	+	-	-	5.1	-
2	thymol	1308	1310	+	0.8	-	-	-
3	eugenol	1339	1344	+	6.0	14.6	51.6	-
4	α-copaene	1381	1387	+	-	6.3	3.7	-
5	β-caryophyllene	1412	1416	+	77.4	77.1	38.6	-
6	isoeugenol	1432	1439	+	-	-	1.0	-
7	α-humulene	1450	1454	+	10.0	0.8	-	-
8	δ-cadinene	1528	1530^+^	+	5.8	1.3	-	-
	Total (%)				100.0	100.0	100.0	

^1^ Compound identification number; ^2^ compounds are reported according their elution order on column; ^3^ linear retention indices measured on apolar columns; ^4^ linear retention indices from literature; ^+^ normal alkane RI; ^5^ identification by MS spectra, tr < 0.1%.

**Table 4:** IC_50_ values of *Harpagophytum procumbens* root extract (HPE) dissolved in DMSO (HPE_DMSO_), 100% *v*/*v* EtOH (HPE_EtOH100_), 50% *v*/*v* EtOH (HPE_EtOH50_), and deionized water (HPE_H2O_) and the positive control JZL 195 in the FAAH inhibition assay.

*Harpagophytum procumbens* Root Extract	IC_50_ (CL) μg/mL
HPE_DMSO_	94.7 (23.8–97.5)
HPE_EtOH100_	65.5 (19.2–87.9) *
HPE_EtOH50_	73.8 (24.5–94.3)
HPE_H2O_	-
JZL 195	0.03 (0.01–0.06) *

Not evaluable being lower than 80% inhibition achieved. * *p* < 0.05 significantly lower than HPE_DMSO_ (ANOVA followed by Bonferroni’s multiple comparison post hoc test).

## Supplementary Materials

The following materials are available online at https://www.mdpi.com/2072-6643/12/9/2545/s1, Figure S1: Effects of all HPE extracts on CB2 receptor and PI-PLC β2 protein production after 48 h treatment.
