# Supplementary material for: Antiarthritic Effects of a Root Extract from Harpagophytum procumbens DC: Novel Insights into the Molecular Mechanisms and Possible Bioactive Phytochemicals
Source: Nutrients. 2020 Aug 23;12(9):2545. doi: 10.3390/nu12092545 (PMC7551290; doi:10.3390/nu12092545)
Supplement: Supplementary file 1 [file nutrients-12-02545-s001.pdf]

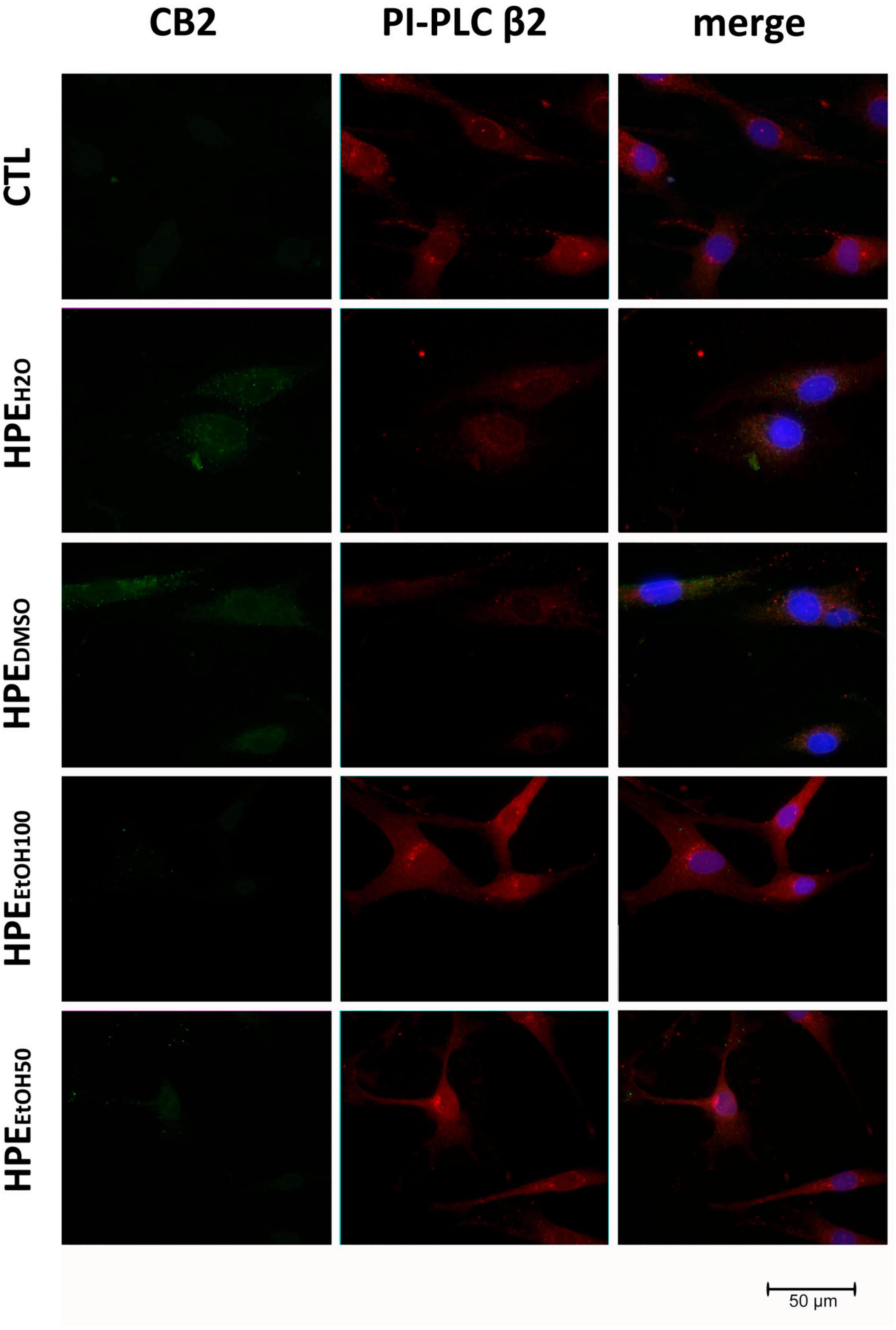
CB2
PI-PLC β2
merge
CTL
HPEH2O
HPEDMSO
HPEEtOH100
HPEEtOH50
50 µm

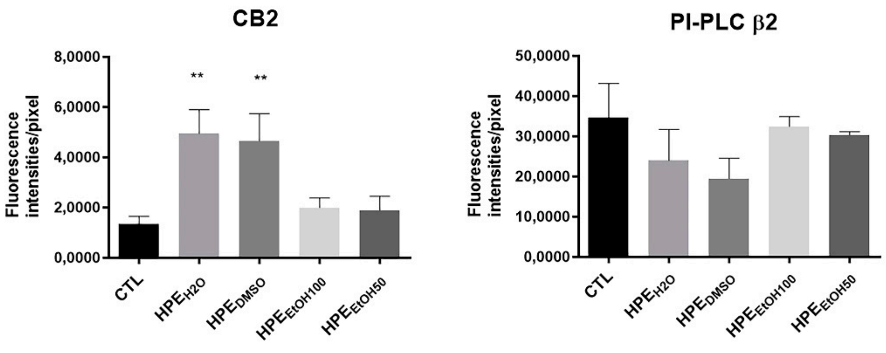


**Figure S1.** Effects of all HPE extracts on CB2 receptor and PI-PLC β2 protein production. Upper panel: cells were treated with 0.1 mg/mL, of *Harpagophytum procumbens* root extract (HPE) dissolved in deionized water ($HPE_{H2O}$), DMSO ($HPE_{DMSO}$), 100 % v/v EtOH ($HPE_{EtOH100}$) and 50 % v/v EtOH ($HPE_{EtOH50}$), for 48 hours and then analyzed by immunofluorescence using anti-CB2 and anti-PI-PLC β2 primary antibodies and Alexa Fluor 488 (green, CB2) and Alexa Fluor 568 (red, PI-PLC β2) secondary antibodies. Nuclei were stained with DAPI, (original magnification 40 x). Lower panel: The sum of all pixel intensities in the region of interest were obtained by ImageJ. ** $p<0,01$
